# *TfWRKY40* positively regulates diosgenin biosynthesis in *Trigonella foenum-graecum* L.

**DOI:** 10.3389/fpls.2025.1666107

**Published:** 2025-09-24

**Authors:** Chuanjia Xu, Nan Tang, Yehan Xu, Changfu Li, Yansheng Zhang

**Affiliations:** ^1^ School of Environmental and Chemical Engineering, Shanghai University, Shanghai, China; ^2^ Shanghai Key Laboratory of Bio-Energy Crops, Synthetic Biology Research Center, School of Life Sciences, Shanghai University, Shanghai, China

**Keywords:** *TfWRKY40*, transcription factor, positive regulator, diosgenin biosynthesis, *Trigonella foenum-graecum*

## Abstract

Diosgenin is a bioactive steroidal natural product extraced from plants and serves as an important precursor for the industrial production of steroidal hormone drugs. Despite its pharmacological significance, the biosynthetic and regulatory mechanisms underlying diosgenin production in the medicinal plant *T. foenum-graecum* remain poorly understood. In this study, we identified *Tf*WRKY40, a WRKY transcription factor from *T. foenum-graecum*, whose expression strongly correlates with diosgenin accumulation. Using RNA interference and overexpression strategies combined with transcriptomic analysis and targeted metabolite quantification, we demonstrated that silencing of *TfWRKY40* led to a 67.60% reduction in diosgenin content, which was accompanied by downregulation of key biosynthetic genes or transcript variants including *ACAT1*, *HMGR1*, *PMK1*, *MVD*, *FPS*, *SQE2*, *CAS1*, *SMO3-1*, *SMO3-2*, *8,7-SI*, *SMO4-3*, *CYP90B50*, and *CYP82J17* in the transgenic hairy roots. Conversely, overexpression of *TfWRKY40* resulted in a 59.25% increase in diosgenin levels, along with upregulation of these biosynthetic genes or transcript variants. Taken together, these findings suggest that *TfWRKY40* acts as a positive regulator of diosgenin biosynthesis in *T. foenum-graecum*, likely by activating the transcription of critical pathway genes, particularly *CAS1*, *HMGR1*, and *CYP90B50*. This work highlights *TfWRKY40* as a promising target for metabolic engineering strategies aimed at enhancing diosgenin production and facilitating the development of diosgenin-derived steroidal therapeutics.

## 1 Introduction

Diosgenin, a steroidal sapogenin widely distributed in both monocotyledonous and dicotyledonous plants, including *Dioscorea zingiberensis* and *Trigonella foenum-graecum*, has emerged as a pharmacologically important plant-derived secondary metabolite with diverse therapeutic potential (Ge et al., 2015; Zhang et al., 2020; Katoch et al., 2024). This bioactive compound exhibits remarkable anti-cancer (He et al., 2014; Meng et al., 2019), anti-inflammatory (Wei et al., 2023), neuroprotective (Man et al., 2025), cardiovascular-protective (Katoch et al., 2024), and lipid-lowering (Xiang et al., 2024) properties, primarily through modulation of cell cycle progression and cellular signaling pathways. Owing to its structural versatility, many diosgenin-derived amino acid conjugates have been synthesized and investigated for their potential to treat pathological conditions such as cancer, diabetes, and neurodegenerative diseases (Huang et al., 2017; Cai et al., 2018; He et al., 2018; Mohseni-Moghaddam et al., 2023). Additionally, diosgenin serves as a critical precursor for the industrial synthesis of steroidal hormone drugs, including testosterone, progesterone, hydrocortisone, and estradiol, underscoring its significance in pharmaceutical applications (Huang et al., 2010; Zhang et al., 2016; Dong et al., 2018).

*T. foenum-graecum* is widely adopted as a model system for diosgenin studies, due to its short growth cycle and ease of cultivation (Ciura et al., 2017a, 2018). Previous studies have demonstrated that exogenous elicitors, including methyl jasmonate (MeJA), cholesterol, and squalene, significantly enhance diosgenin accumulation in *T. foenum-graecum* (Chaudhary et al., 2015; Ciura et al., 2017a, 2018). These efforts have led to two major breakthroughs: the identification of critical biosynthetic enzymes, especially members of the CYP72A family as essential components, in steroidal saponin biosynthesis (Chaudhary et al., 2015), and the characterization of diosgenin accumulation dynamics under various elicitor treatments (Ciura et al., 2017a, 2018). Diverse germplasm screening has also identified high-yielding lines, laying the foundation for future metabolic engineering and crop improvement initiatives (Dwivedi et al., 2017; Coban et al., 2025).

Diosgenin biosynthesis in plants originates from cholesterol (Joly et al., 1969a; 1969b; Stohs et al., 1969), which itself is synthesized from the C5 isoprenoid precursors isopentenyl pyrophosphate (IPP) and dimethylallyl pyrophosphate (DMAPP). Higher plants produce IPP and DMAPP primarily through the cytosolic mevalonate (MVA) pathway, which is derived from acetyl-CoA with the 3-hydroxy-3-methylglutaryl-coenzyme A reductase (HMGR) acting as a rate-limiting enzyme in this process. IPP is subsequently converted to 2,3-oxidosqualene. The biosynthetic cascade begins with the cyclization of 2,3-oxidosqualene to cycloartenol, catalyzed by cycloartenol synthase (CAS), the precursor to cholesterol formation. Multi-omics analyses in *T. foenum-graecum* leaves have confirmed that only CAS is expressed in leaves, the primary diosgenin-producing tissue, while lanosterol synthase (LAS) activity is absent, thereby validating cycloartenol as a key biosynthetic intermediate (Mohammadi et al., 2020). Stable isotope tracing has further substantiated cholesterol as the direct precursor of diosgenin and elucidated the complete nine-step enzymatic conversion from cycloartenol to cholesterol, including these enzymes such as CAS, sterol side chain reductase 2 (SSR2), C4-sterol methyl oxidase (SMO), cyclopropylsterol isomerase (CPI), CYP51, sterol C-14 reductase (C14-R), sterol 8,7 isomerase (8,7-SI), sterol C-5(6) desaturase 2 (C5-SD2) and 7-dehydrocholesterol reductase 2 (7-DR2) (Sonawane et al., 2017). Seminal studies by Sawai et al. (2014) identified SSR2 and C5-SD2 as critical genes in the cholesterol biosynthesis pathway in *Solanaceae* plants, catalyzing the conversion of cycloartenol to cycloartanol and cholesta-7-en-3*β*-ol to 7-dehydrocholesterol, respectively. In the process of converting cholesterol to diosgenin, cholesterol undergoes site-specific oxidations at the C-22, C-16, and C-26 positions (Christ et al., 2019; Zhou et al., 2022). Transcriptome analyses of methyl jasmonate-treated *T. foenum-graecum* seedlings identified cytochrome P450s (CYPs) and UDP-glycosyltransferases (UGTs) as key enzymes responsible for these modifications (Zhou et al., 2019). Two functional CYPs including *TfCYP90B50* and *TfCYP72A613*/*TfCYP82J17* have been characterized (Christ et al., 2019); however, due to the structural complexity of steroidal sapogenins, the complete biosynthetic and regulatory pathways remain unknown.

WRKY transcription factors, one of the ten major plant transcription factor families, are increasingly recognized as key regulators of secondary metabolite biosynthesis (Rushton et al., 2010; Schluttenhofer and Yuan, 2015). In *Withania somnifera*, for instance, *Ws*WRKY1 regulates withanolide biosynthesis by modulating the expression of squalene synthase (*SQS*) and squalene epoxidase (*SQE*) genes (Singh et al., 2017). In *Artemisia annua*, *AaWRKY1* promotes artemisinin production by directly regulating the transcription of the *CYP71AV1* gene (Han et al., 2014). In *Dioscorea composita*, *Dc*WRKY11 acts as a positive regulator in the biosynthesis of steroidal saponins (Yu et al., 2024). Similarly, *Pg*WRKY4X from *Panax ginseng* binds to the W-box elements in the promoter of *SQE* gene, thereby enhancing ginsenoside biosynthesis (Yao et al., 2020). Other WRKY transcription factors, such as *Pq*WRKY1 in *P. quinquefolius* (Sun et al., 2013), and *Cb*WRKY24 in *Conyza blinii* (Sun et al., 2018), act as positive regulators in response to MeJA, upregulating key genes in the MVA pathway, thereby promoting the biosynthesis of triterpene ginsenoside and blinin, respectively. Additionally, the interactions between the WRKY transcription factors and miRNAs have been implicated in regulating triterpenoid saponin accumulation in *Sapindus mukorossi* (Xu et al., 2023). Collectively, these studies underscore the pivotal role of WRKY transcription factors in modulating complex secondary metabolic pathways, especially those associated with sterol-derived and saponin-type compounds.

In this study, we performed comparative transcriptome analysis of *T. foenum-graecum* seedlings treated with MeJA to identify transcriptional regulators involved in diosgenin biosynthesis. Through this approach, we isolated a WRKY transcription factor, designated *TfWRKY40*, from the MeJA-treated *T. foenum-graecum* seedlings. Phylogenetic tree analysis and *Agrobacterium rhizogenes*-mediated hairy root transformation were then used to functionally characterize *TfWRKY40* and investigate its positive regulatory role in diosgenin biosynthesis. Moreover, comparative transcriptomic and metabolite analyses indicated that *TfWRKY40* likely activates the expression of key biosynthetic genes or transcript variants, particularly *HMGR1*, *CAS1*, and *CYP90B50*, thereby regulating diosgenin biosynthesis. These findings not only identify *TfWRKY40* as a positive regulator of diosgenin biosynthesis but also provide new insights into the regulatory mechanisms controlling cholesterol-derived secondary metabolism in plants. These results have potential applications in metabolic engineering strategies aimed at enhancing the biosynthesis of pharmacologically important steroidal compounds.

## 2 Materials and methods

### 2.1 Plant materials and growth conditions

*T. foenum-graecum* seeds used in this study were collected from Shandong Province, China, and *Nicotiana benthamiana* seeds were provided by the laboratory of Professor Xiangyang Hu at the Shanghai University of China. All plant materials were cultivated in a controlled climate chamber under the following conditions: temperature of 25°C ± 2°C, a photoperiod of 16 hours light/8 hours dark, and relative humidity of 60%-70%.

### 2.2 MeJA treatment of *T. foenum-graecum* seedlings

The *T. foenum-graecum* seeds with consistent size were carefully selected and initially soaked in 75% ethanol for 1 minute. Subsequently, the seeds were rinsed three times with sterile distilled water. Surface sterilization was then performed by immersing the seeds in a NaClO solution containing 5%~10% (w/v) available chlorine for 10 minutes, followed by three additional rinses with sterile distilled water to remove any residual disinfectant. The sterilized seeds were placed on 0.65% (w/v) water-agar medium and incubated in darkness at 25 ± 2°C for 42–44 hours.

MeJA was dissolved in absolute ethanol and then added to half-strength Murashige and Skoog (1/2 MS) liquid medium to obtain a final ethanol concentration of 0.01% (v/v). The control medium contained absolute ethanol at the same final concentration without MeJA. Both the MeJA-supplemented and control media were dispensed into sterile culture bottles (35 mL per bottle), which were then filled with sterile glass beads (approximately 0.5 cm in diameter) until the beads slightly protruded above the liquid surface.

Germinated seedlings were transferred into the MeJA-supplemented and control media, with their roots being gently secured by sterile glass beads (approximately 25 seedlings per bottle). The cultures were maintained under a 16-hour light/8-hour dark photoperiod at 25 ± 2°C. *T. foenum-graecum* seedlings were harvested at 6, 12, 24, 48, 72, and 120 hours following the MeJA treatment.

### 2.3 Extraction and quantitative analysis of diosgenin from *T. foenum-graecum* plants

*T. foenum-graecum* seedlings or hairy roots were ground to a fine powder under liquid nitrogen, and the resulting powder was dried to a constant weight at 37°C. Twenty milligrams of the dried powder were accurately weighed and extracted with 1 mL of methanol containing 20 μg of ursolic acid as an internal standard. Extraction was conducted using an ultrasonic bath and repeated three times. The pooled extracts were then evaporated to dryness under a gentle stream of nitrogen gas at ambient temperature. The residue was resuspended in distilled water, and concentrated sulfuric acid was added to achieve a final concentration of 1.8 M. Hydrolysis was performed by incubating the mixture in a 95°C water bath for 10–12 hours. After hydrolysis, liquid-liquid extraction was carried out by vigorous shaking twice with n-hexane, followed by a subsequent extraction with ethyl acetate. The organic layers were combined, evaporated to dryness under nitrogen, and the resulting residue was reconstituted in 100 μL methanol for subsequent HPLC analysis.

Diosgenin content was determined using a Shimadzu LC-20AT high-performance liquid chromatography (HPLC) system equipped with a Shim-pack GIST C18 column (250 mm × 4.6 mm, 5 μm particle size). The column temperature was maintained at 37°C. The mobile phase consisted of ultrapure water (aqueous phase) and HPLC-grade acetonitrile (organic phase) mixed at a ratio of 1:9 (v/v). Isocratic elution was performed at a flow rate of 0.8 mL/min with a total run time of 40 minutes. Detection was conducted using a UV detector set at 203 nm. The Shimadzu LabSolutions software (version 5.106 SP1) was used for the integration of targeted peaks and quantification of corresponding metabolites. Representative chromatograms of diosgenin and the internal standard ursolic acid in both chemical standards and sample extracts are provided in **Supplementary Figure S1**.

For the quantitative analysis, the following formula was used:


$$f=\frac{As}{Cs}\times \frac{Cr}{Ar},\;Cx=f\times Ax\times \frac{Cs^{\prime }}{As^{\prime }}$$

where f is the correction factor; As and Cs represent the peak area and concentration of the internal standard (ursolic acid), respectively; Ar and Cr are the peak area and concentration of the reference standard (diosgenin); Ax ​ and Cx refer to the peak area and concentration of diosgenin in the sample, respectively; $\;As^{\prime }$ ​ is the peak area of the internal standard (ursolic acid) in the sample, and $Cs^{\prime }$ is the concentration of the internal standard added to the sample.

### 2.4 RNA extraction and quantitative real-time PCR

Total RNA (1 μg) was extracted using the EASYspin Plus Plant RNA Rapid Extraction Kit (Aidlab, Beijing, China), and the first-strand cDNA was synthesized using the TransScript^®^ One-Step gDNA Removal and cDNA Synthesis SuperMix Kit (TransGen Biotech, China), following the manufacturers’ protocols. Quantitative real-time PCR (qRT-PCR) was performed using the Taq Pro Universal SYBR qPCR Master Mix (Vazyme Biotech, Nanjing, China) on a Bio-Rad CFX96™ Real-Time PCR Detection System (Bio-Rad, Hercules, CA, USA), according to the manufacturer’s instructions.

The thermal cycling conditions were as follows: initial denaturation at 95°C for 10 minutes; followed by 40 cycles of denaturation at 95°C for 15 seconds, annealing at 58°C for 40 seconds, and extension at 72°C for 30 seconds. Following the amplification, quantification cycle (Cq) values were recorded and used for statistical analysis to determine the relative expression levels of the genes of interest. All qRT-PCR assays were performed with three biological replicates, each analyzed in three technical replicates. Relative gene expression levels were calculated using the 2^−ΔΔCT^ method.

### 2.5 Phylogenetic tree construction and multiple sequence alignments

Sequence data were retrieved from the NCBI database (https://www.ncbi.nlm.nih.gov/). Phylogenetic trees were constructed employing the neighbor-joining method in MEGA software (version 7.0.26), with 2000 bootstrap replications for reliability. Sequence alignments were conducted using the DNAMAN software (version 9.0.1.116).

### 2.6 Subcellular localization

To analyze the subcellular localization of the *Tf*WRKY40 protein, the full-length coding sequence excluding stop codon was amplified and cloned into the *Bam*HI and *Sac*I sites of the pHB-GFP (enhanced green fluorescent protein, EGFP) expression vector, generating the fusion construct pHB*-TfWRKY40-*GFP. The empty vector pHB-GFP was used as a negative control. The recombinant plasmids were co-transformed with the nuclear-localized marker pRI101-H2B-CFP (enhanced cyan fluorescent protein, ECFP) into *A. tumefaciens* strain GV3101. The resulting *A. rhizogenes* suspensions were infiltrated into the leaves of *N. benthamiana*.

Fluorescence signals were observed 48-72 hours post-infiltration using a ZEISS two-photon laser scanning confocal microscope. EGFP fluorescence was excited at 488 nm and ECFP at 458 nm. Subcellular localization of the *Tf*WRKY40 protein was determined based on the co-localization patterns of EGFP and ECFP signals. The experiment was performed with three independent biological replicates, and at least 10 microscopic fields were examined for each sample.

### 2.7 Plant expression vector construction and *A. tumefaciens*-mediated transformation of *T. foenum-graecum* hairy roots

To construct the plant overexpression vector for *TfWRKY40*, the coding sequence of *TfWRKY40* was amplified using the primers *TfWRKY40*-OE-F and *TfWRKY40*-OE-R. The amplified fragment was inserted into the *Bam*HI and *Sac*I sites of the plant expression vector pBI121. For gene silencing, the RNA interference (RNAi) vector pK7GWIWG2_II-RedRoot was used to suppress *TfWRKY40* expression. A 273-bp fragment of the *TfWRKY40* coding region was amplified by PCR using the primers *TfWRKY40*-RNAi-F and *TfWRKY40*-RNAi-R. The PCR product was subsequently introduced into the pDONR201 vector via BP recombination, followed by LR recombination into the pK7GWIWG2_II-RedRoot vector.

The resulting plant expression constructs, along with their corresponding empty vectors as negative controls, were transformed into *A. rhizogenes* strain Ar.Qual using a freeze-thaw method. Hairy roots were induced following the protocol described by (Garagounis et al., 2020), with minor modifications. In brief, *T. foenum-graecum* seeds were surface-sterilized by immersion in 70% ethanol for 1 min, followed by three washes with sterile distilled water. Seeds were then sterilized in a NaClO solution containing 5%~10% (w/v) available chlorine for 10 min, followed by three washes with sterile water. The sterilized seeds were germinated on 0.65% (w/v) agar plates in the dark at 24°C for 36 h. Germinated seedlings were then used for transformation. The radicles of the seedlings were excised, and the injured surfaces were immersed in the *Agrobacterium* slurry harboring the target constructs for 3–5 min. The inoculated seedlings were transferred onto large square petri dishes containing solid 1/2 MS medium. Plates were placed in a growth chamber at 22°C under a 16 h light/8 h dark photoperiod. Callus formation at the infected sites was observed approximately 2 weeks post-inoculation, and hairy root emergence occurred around 3 weeks post-inoculation. Approximately 40–50 days later, transgenic roots were confirmed by qRT-PCR and used for metabolite analysis. Each biological replicate consisted of pooled hairy roots from 30 infected plants. Data for each construct were collected from at least three independent biological replicates. All primers used in this study are listed in **Supplementary Table S1**.

### 2.8 RNA-seq library preparation and sequencing

Total RNA was extracted from the transformed *T. foenum-graecum* hairy roots, and submitted to Novogene Co., Ltd (Beijing, China) for high-throughput sequencing. RNA integrity and purity were first verified using Agilent Bioanalyzer 2100 (Agilent Technologies, Palo Alto, CA, USA) before library construction. Poly(A)+ mRNA was enriched from total RNA using oligo(dT)-coupled magnetic beads, followed by fragmentation under optimized conditions. Double-stranded cDNA was synthesized from the fragmented mRNA, subjected to end repair and A-tailing, and then ligated with Illumina sequencing adapters. The resulting libraries were size-selected (300–500 bp), PCR-amplified, and rigorously quality-checked before being sequenced on the Illumina HiSeq 2000 platform (San Diego, CA, USA). Raw sequencing reads were processed to generate high-quality clean reads by removing adapter sequences, the reads containing more than 10% ambiguous bases (N), and the low-quality reads in which more than 50% of the bases had Phred scores ≤ 20. Three biological replicates were used for RNA sequencing. For each biological replicate, total RNA was extracted from a pool of hairy roots derived from 30 independently infected plants.

### 2.9 Transcriptome analysis

Transcript assembly was performed using the Trinity software. To evaluate transcript abundance, clean reads were individually mapped to the assembled transcriptome, and transcript abundance for each unigene was normalized using the FPKM (fragments per kilobase per million mapped reads) method (Trapnell et al., 2010). The annotated unigenes were subsequently queried against the Gene Ontology (GO) database (http://www.geneontology.org/) and the Kyoto Encyclopedia of Genes and Genomes KEGG (http://www.genome.jp/kegg/). Functional annotation and enrichment analysis of differentially expressed genes (DEGs) were conducted using KEGG (Kanehisa and Goto, 2000). DEGs were identified using the DESeq2 R package, with a threshold of fold change (FC) ≥ 2 and False Discovery Rate (FDR)< 0.05 (Storey and Tibshirani, 2003; Anders and Huber, 2010; Love et al., 2014).

### 2.10 Statistical analysis

All experiments were performed with at least three biological replicates. Data were analyzed using GraphPad Prism 9 software (Version 9.4.1) and expressed as mean ± standard deviation (mean ± SD). Statistical significance was determined by a Student’s *t*-test, with ***p< 0.001, **p< 0.01, and *p< 0.05 being considered statistically significant.

## 3 Results

### 3.1 *TfWRKY40* closely correlates to diosgenin biosynthesis in *T. foenum-graecum*

**Figure 1:**
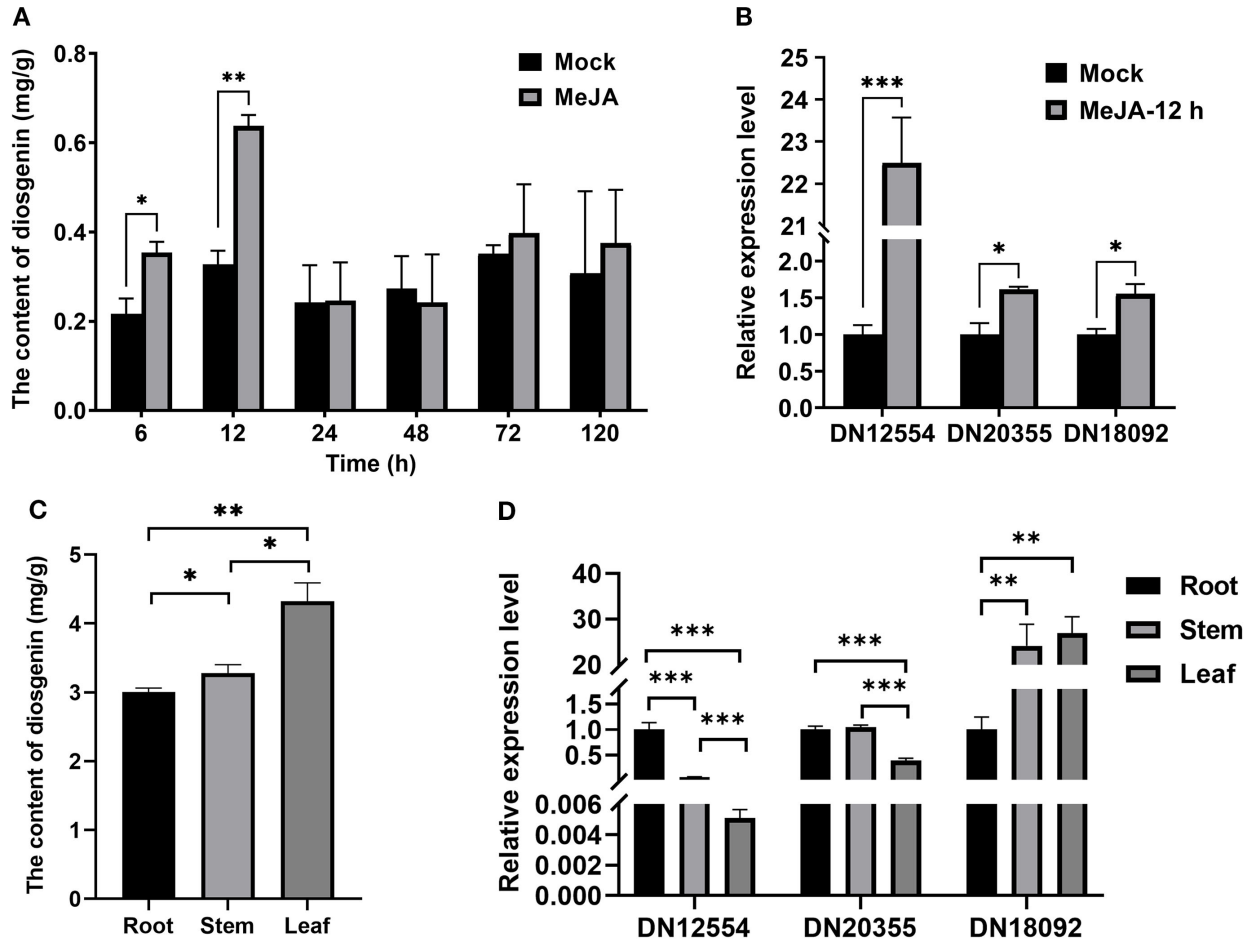
Correlation between DN18092 (*TfWRKY40*) expression and diosgenin biosynthesis in *T. foenum-graecum*. **(A)** Methyl jasmonate (MeJA) treatment significantly increased diosgenin accumulation at 6 h and 12 h compared to mock-treated controls. **(B)** Quantitative RT-PCR analysis showed that 12-h MeJA treatment markedly upregulated three transcription factor genes, identified from our previously published transcriptome data, which are implicated in diosgenin biosynthesis. **(C)** Tissue-specific distribution of diosgenin in *T. foenum-graecum*. Diosgenin content was quantified by HPLC in roots, stems, and leaves of 3-month-old plants. **(D)** Among the three transcription factors, only DN18092 expression levels showed a correlation with diosgenin accumulation in the above-mentioned tissues of *T. foenum-graecum*. Data represent mean ± SD (*n*=3 biological replicates). Statistical significance was determined by Student’s *t*-test (*p< 0.05, **p< 0.01, ***p< 0.001). Gene expression was normalized to *TfActin* and expressed as fold-change relative to controls.

To assess the temporal effects of MeJA on diosgenin accumulation, *T. foenum-graecum* seedlings were treated with 0.01% (v/v) MeJA and harvested at 6, 12, 24, 48, 72, and 120 hours post-treatment for diosgenin extraction and quantification. Diosgenin content ranged from 0.22 to 0.64 mg/g dry weight (DW) across all time points, peaking at 12 hours post-treatment (0.64 mg/g DW) (**Figure 1A**). During the early phase (6–12 h), MeJA treatment significantly increased diosgenin levels, reaching 1.63- and 1.95-fold relative to their respective controls at 6 and 12 hours, respectively (**Figure 1A**). No significant induction was detected during the mid-phase (24–48 h), with a slight decrease observed in some samples. In the late phase (72–120 h), a modest upward trend was observed, although the changes were not statistically significant (**Figure 1A**). These results suggest that MeJA transiently but robustly activates diosgenin biosynthesis, with a maximal accumulation at 12 hours post-treatment.

To identify the transcriptional regulators involved in this response, we performed a combined analysis of our previously constructed MeJA-responsive transcriptome (Zhou et al., 2019) and the publicly available transcriptome data from different *T. foenum-graecum* tissues (Christ et al., 2019). From an initial set of 4,499 transcription factor-annotated unigenes, 16 transcription factors were considered as potential candidates, as their expression was consistent with diosgenin accumulation in a MeJA-induced or tissue-specific manner. Comparative transcriptomic analysis further narrowed these 16 candidates to three genes (*DN18092_c0_g1*, *DN20355_c0_g1*, and *DN12554_c0_g1*), hereafter referred to as *DN18092*, *DN20355*, and *DN12554*, respectively, based on putative functional annotation (Christ et al., 2019; Zhou et al., 2019). Importantly, *DN18092* emerged as the most promising candidate, showing significant upregulation (log2 fold change > 2, *p*< 0.01) under MeJA treatment (Zhou et al., 2019).

qRT-PCR was performed to investigate the expression levels of *DN18092*, *DN20355*, and *DN12554* in *T. foenum-graecum* seedlings treated with MeJA for 12 hours, corresponding to the peak of diosgenin accumulation. Results showed that the expression of all three genes was significantly upregulated compared to the mock controls (**Figure 1B**), which correlated with the increased diosgenin content in the same samples (**Figure 1A**). We then analyzed the tissue-specific expression of *DN18092*, *DN20355*, and *DN12554* in *T. foenum-graecum*. Diosgenin was found to accumulate at significantly higher levels in above-ground organs (i.e., leaves and stems) than in roots (**Figure 1C**). Similarly, *DN18092* transcript levels were higher in leaves and stems than in roots (**Figure 1D**), consistent with the tissue-specific distribution of diosgenin. In contrast, *DN20355* and *DN12554* showed relatively higher expression in roots, with the lowest expression detected in leaves. Taken together, *DN18092* showed a positive correlation with diosgenin biosynthesis in both MeJA-induced and tissue-specific expression patterns, and was therefore selected for further investigation.

**Figure 2:**
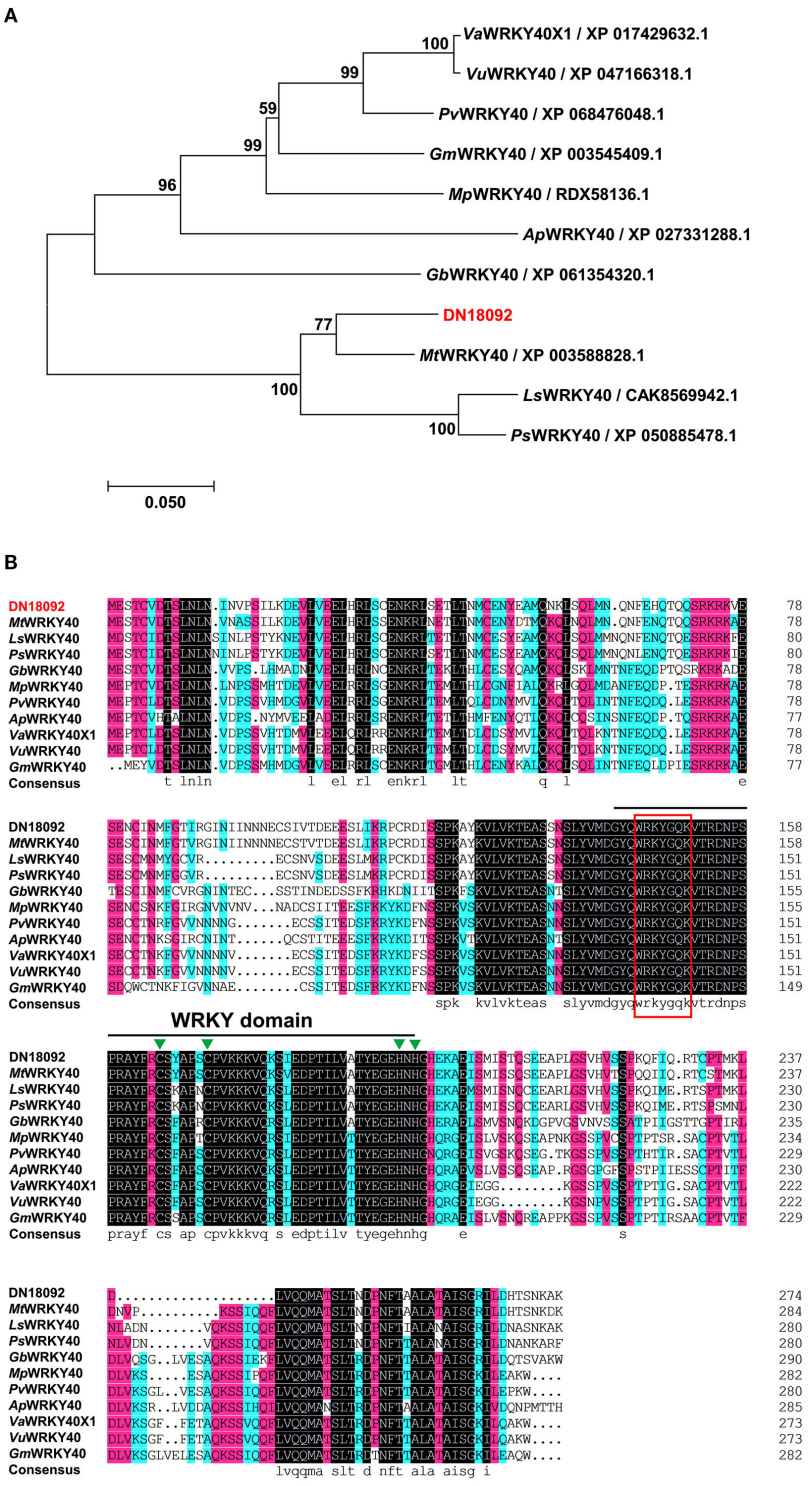
Phylogenetic and sequence analysis of WRKY40 proteins. **(A)** A neighbor-joining phylogenetic tree of *Tf*WRKY40 and its orthologs from *Medicago truncatula*, *Lathyrus sativus*, *Pisum sativum*, *Gastrolobium bilobum*, *Mucuna pruriens*, *Phaseolus vulgaris*, *Abrus precatorius*, *Vigna angularis*, *Vigna umbellata* and *Glycine max* was constructed using a MEGA software (version 7.0.26). The *Tf*WRKY40 is highlighted in red. **(B)** Multiple sequence alignment of *Tf*WRKY40 with its orthologs from the selected legume species was performed using a DNAMAN (version 9.0.1.116) software. The WRKY domain and the conserved WRKY sequence are indicated by a black line and a red box, respectively. The C2H2-type zinc finger motif is marked with a green triangle.

**Figure 3:**
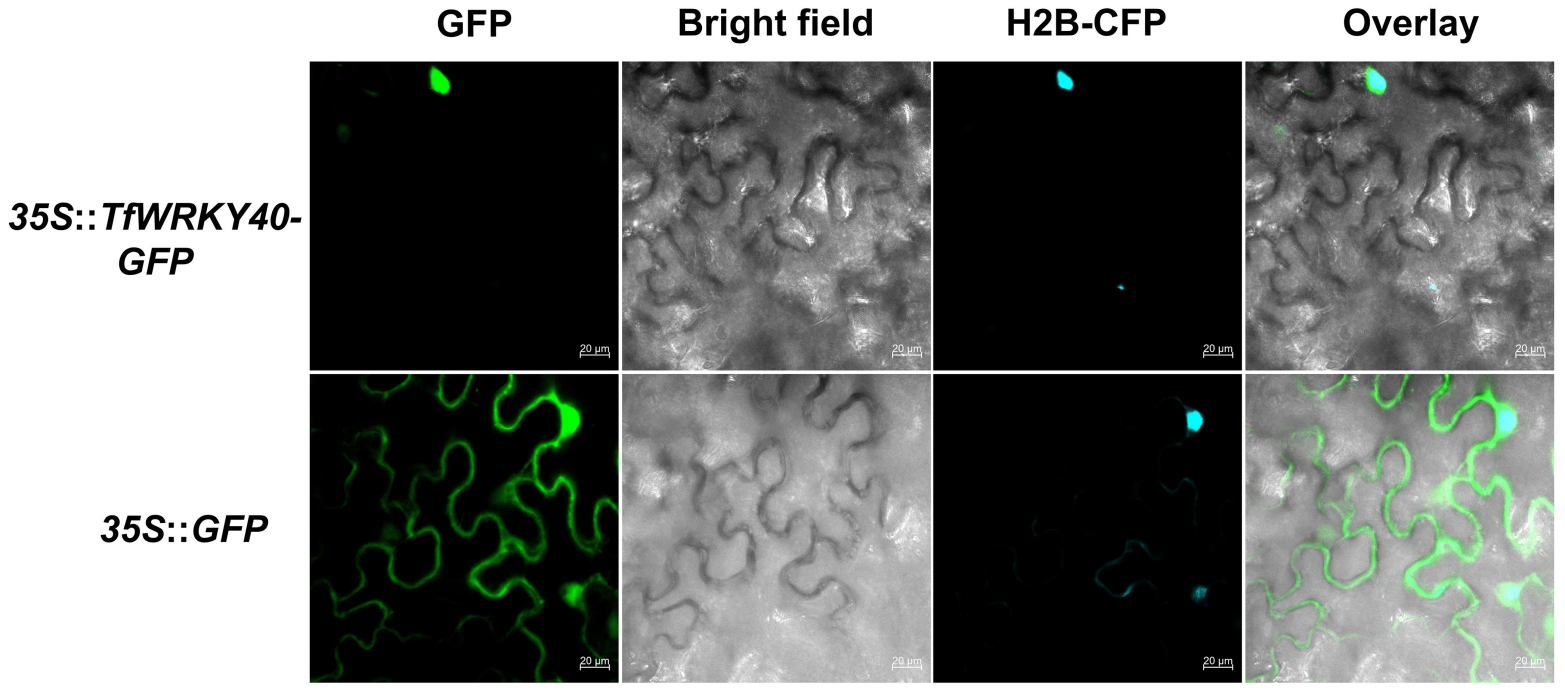
*Tf*WRKY40 localizes to the nucleus in *N. benthamiana* leaf epidermal cells. The *35S*::*TfWRKY40*-*EGFP* fusion construct and nuclear marker *H2B*-*ECFP* containing a chromatin-binding histone tag were co-transformed into leaf epidermal cells, with *35S*::*GFP* as negative control. Fluorescence microscopy demonstrated the nuclear localization of *Tf*WRKY40. Scale bar = 20 μm.

Phylogenetic analysis revealed that DN18092 shares high homology with the WRKY family members, clustering closest to *Mt*WRKY40 from *Medicago truncatula* (**Figure 2A**). Protein sequence alignment confirmed the presence of the conserved WRKY domain and C2H2 zinc finger motif characteristic of this family (**Figure 2B**). Accordingly, DN18092 was named *Tf*WRKY40. Subcellular localization analysis was performed by transient expression of *TfWRKY40-GFP* fusion protein in *N. benthamiana* leaves. Confocal microscopy revealed that *Tf*WRKY40-GFP co-localized with the nuclear marker H2B-ECFP, while free GFP control exhibited diffuse fluorescence throughout the cell (**Figure 3**). These results confirm nuclear localization of *Tf*WRKY40, consistent with its predicted function as a transcription factor.

### 3.2 *Tf*WRKY40 is a positive regulator in diosgenin biosynthesis

To investigate whether *TfWRKY40* plays a regulatory role in diosgenin biosynthesis in *T. foenum graecum*, the overexpression and RNAi constructs targeting *TfWRKY40*, designated pBI121-*TfWRKY40* (*TfWRKY40*-OE) and pK7GWIWG2D-Redroot-*TfWRKY40* (*TfWRKY40*-RNAi), were prepared using the Gateway recombination method. These plant expression constructs, as well as their corresponding empty vectors, were introduced into *T. foenum graecum* hairy roots via *Agrobacterium rhizogenes*-mediated transformation. The transgenic hairy roots were then harvested for gene expression analysis and diosgenin content measurement.

**Figure 4:**
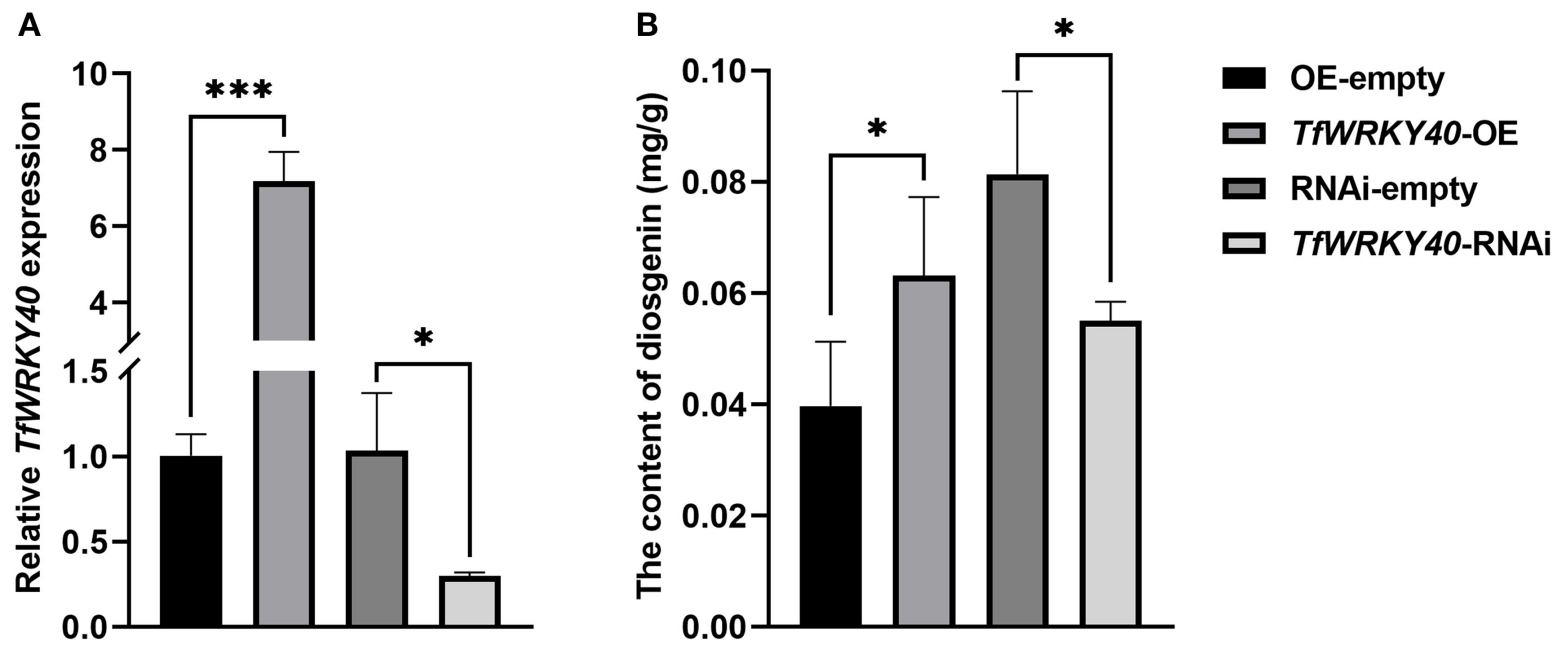
*Tf*WRKY40 is a positive regulator for diosgenin biosynthesis in *T. foenum-graecum*. Analysis of *TfWRKY40* expression **(A)** and diosgenin contents **(B)** in the transgenic *T. foenum-graecum* hairy roots. The hairy roots transformed with either pBI121-*TfWRKY40* (*TfWRKY40*-OE) or pK7GWIWG2D-Redroot-*TfWRKY40* (*TfWRKY40*-RNAi) were compared with their corresponding empty vector controls: pBI121 (OE-empty) and pK7GWIWG2D-Redroot (RNAi-empty). Data represent mean ± SD of three biological replicates. Statistical significance was determined by Student's t-test (*p < 0.05; ***p < 0.001).

qRT-PCR analysis revealed that the expression level of *TfWRKY40* was upregulated by 6.15-fold in the *TfWRKY40*-OE transgenic hairy roots and downregulated by 71.24% in the *TfWRKY40*-RNAi lines, compared with their respective empty vector-transformed controls (**Figure 4A**). Overexpression of *TfWRKY40* led to a diosgenin content of 0.063 mg/g in hairy roots, which was 59.25% higher than that of the control (**Figure 4B**). In contrast, silencing of *TfWRKY40* resulted in a 67.60% reduction in diosgenin content in *TfWRKY40*-RNAi hairy roots compared to the control lines (**Figure 4B**). These results demonstrate that *Tf*WRKY40 acts as a positive regulator of diosgenin biosynthesis in *T. foenum-graecum*. Together with prior evidence of its nuclear localization (**Figure 3**), these findings suggest that *TfWRKY40* functions as a nuclear-localized transcriptional activator regulating diosgenin biosynthesis in *T. foenum-graecum*.

### 3.3 *TfWRKY40* transcriptionally regulates diosgenin biosynthesis-related gene expression

To further elucidate the molecular mechanism by which *TfWRKY40* regulates diosgenin biosynthesis, RNA sequencing was performed on the *TfWRKY40*-OE and *TfWRKY40*-RNAi hairy roots, along with their corresponding empty vector controls. Differentially expressed genes were defined using a threshold of FC ≥ 2 and FDR< 0.05. A total of 662 DEGs were identified between the *TfWRKY40*-RNAi and its corresponding empty vector-transformed hairy roots, while 413 DEGs were found in the *TfWRKY40*-OE transgenic hairy roots compared to the empty vector controls. Among these, 41 DEGs were commonly responsive to both overexpression and silencing of *TfWRKY40* in hairy roots (**Supplementary Figure S2**).

GO enrichment analysis revealed that the most significantly enriched molecular functions were “oxidoreductase activity” and “transition metal ion binding,” with each being represented by over 100 DEGs (**Supplementary Figure S3**). Both functions are directly involved in plant redox metabolism. Notably, in *T. foenum-graecum*, diosgenin is synthesized from cholesterol through multiple redox reactions (Christ et al., 2019; Zhou et al., 2022), suggesting *TfWRKY40* may play an important role in the downstream pathway of converting cholesterol to diosgenin.

KEGG pathway analysis showed a predominant enrichment in phenylpropanoid biosynthesis and carbon fixation pathways in photosynthetic organisms, both of which are associated with secondary metabolite production and stress responses (**Supplementary Figure S4**). Diosgenin also functions as a defensive compound in response to environmental stresses, such as pathogen infections and attacks by pests or herbivores (Moses et al., 2014; Ebrahimibasabi et al., 2020; Cheng et al., 2021). These findings indicate that *TfWRKY40* may enhance the ability of *T. foenum-graecum* to cope with environmental stresses through the regulation of diosgenin biosynthesis.

**Figure 5:**
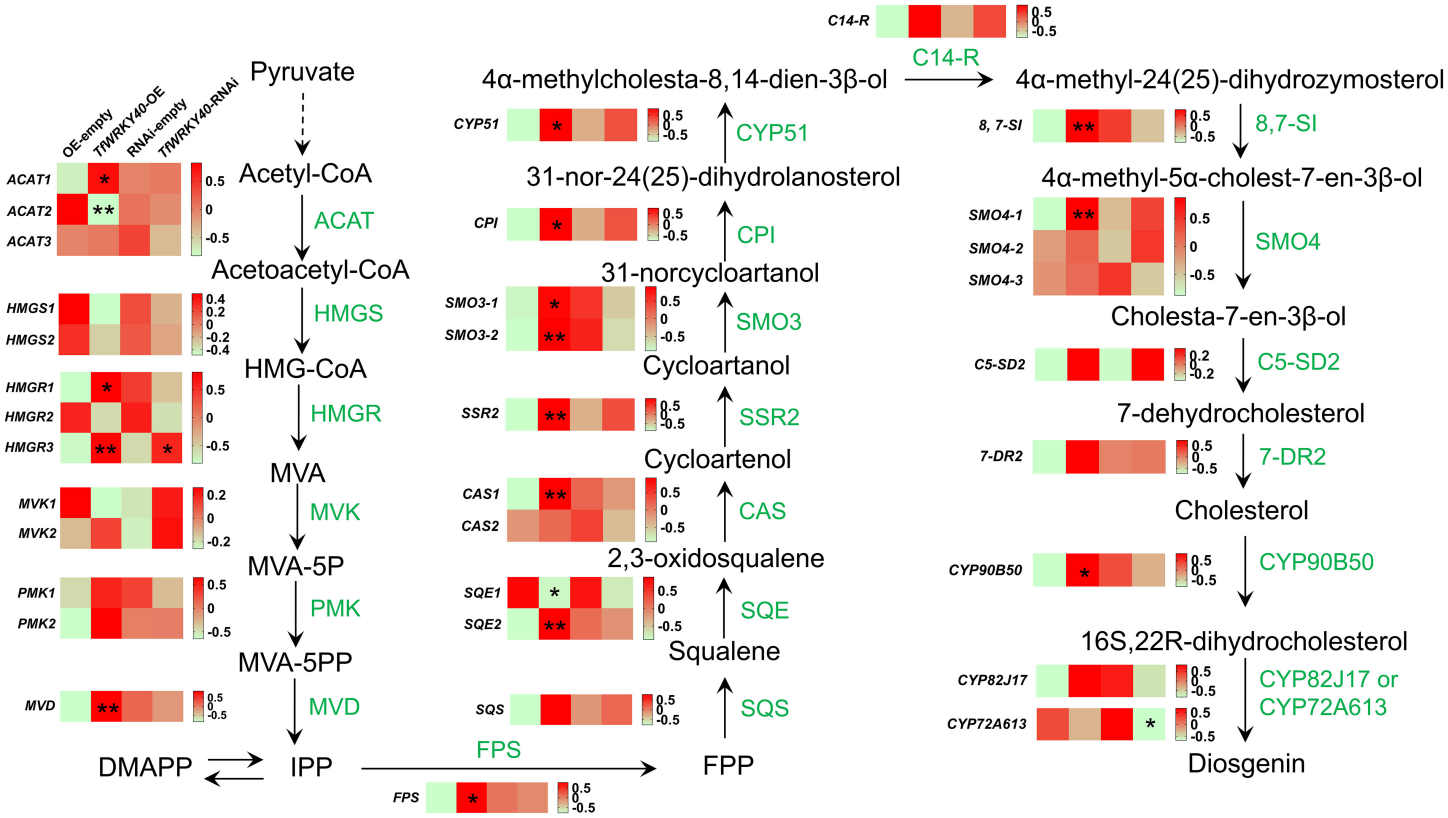
Comparative transcriptomic analysis of the genes involved in the diosgenin biosynthetic pathway. Heat maps display the expression profiles of diosgenin synthesis-related genes or transcript variants in OE-empty, *TfWRKY40*-OE, RNAi-empty, and *TfWRKY40*-RNAi *T. foenum-graecum* hairy roots. Gene expression levels (in FPKM) were log_2_-transformed after adding a pseudocount of 1 (log_2_(FPKM+1)) to stabilize variance and account for zero values. The data were then standardized as z-scores (mean-centered and scaled by standard deviation per gene) and visualized using a color gradient (pale green: low expression; red: high expression). Statistically significant differences compared to the corresponding control (OE-empty or RNAi-empty) are indicated by asterisks on the heatmap blocks: *P*< 0.05 (*) and *P*< 0.01 (**). Heat maps were generated using GraphPad Prism (version 9.4.1). Source data are provided in **Supplementary Table S2**, and the sequences for all the analyzed genes or transcripts are available in **Supplementary Table S3**. ACAT, Acetyl-CoA acetyltransferase; HMGS, hydroxymethylglutaryl-CoA synthase; HMGR, 3-hydroxy-3-methylglutaryl-coenzyme A (CoA); MVK, mevalonate kinase; PMK, phosphomevalonate kinase; MVD, mevalonate dipyrophosphate decarboxylase; FPS, farnesyl pyrophosphate synthase; SQS, squalene synthase; SQE, squalene epoxidase; CAS, cycloartenol synthase; SSR2, sterol side chain reductase 2; SMO, C4-sterol methyl oxidase; CPI, cyclopropylsterol isomerase; C14-R, sterol C-14 reductase; 8,7-SI, sterol 8,7 isomerase; C5-SD2, sterol C-5(6) desaturase 2; 7-DR2, 7-dehydrocholesterol reductase 2; HMG-CoA, 3-Hydroxy-3-methylglutaryl-CoA; MVA, mevalonate; IPP, isopentenyl pyrophosphate; DMAPP, dimethylallyl pyrophosphate; FPP, farnesyl pyrophosphate.

As previously established, the biosynthetic pathway from 2,3-oxidosqualene to cholesterol in plants has been well characterized, involving nine key enzymes, including CAS, SSR2, SMO, CPI, CYP51, C14-R, 8,7-SI, C5-SD2, and 7-DR2 (**Figure 5**). Although the complete biosynthetic pathway from cholesterol to diosgenin remains to be fully elucidated, several crucial P450-encoding genes (*CYP90B50*, *CYP82J17*, and *CYP72A613*) have been identified as crucial components in diosgenin biosynthesis (Christ et al., 2019; Zhou et al., 2022). Therefore, investigating the expression patterns of these key diosgenin biosynthetic genes through transcriptome analysis is pivotal for understanding the regulatory mechanism of *TfWRKY40*.

Gene expression was quantified using normalized FPKM values to assess the changes in the expression of diosgenin biosynthetic genes in both *TfWRKY40*-OE and -RNAi transgenic hairy roots, compared to their respective controls (**Figure 5**; **Supplementary Table S2**). Notably, thirteen transcripts exhibited coordinated transcript level changes in response to both up- and down-regulation of *TfWRKY40* (**Figure 5**), suggesting they are regulated by this transcription factor. These include two transcripts from putative transcript variants or duplicated genes (*ACAT1* and *HMGR1*) in the upstream MVA pathway, nine transcripts (*PMK1*, *MVD*, *FPS*, *SQE2*, *CAS1*, *8,7-SI*, and *SMO3-1*, *SMO3-2* and *SMO4-3*) involved in cholesterol biosynthesis, and two cytochrome P450 genes (*CYP90B50* and *CYP82J17*) associated with cholesterol-to-diosgenin conversion. Among them, HMGR functions as the key rate-limiting enzyme in the MVA pathway (Venkateshwaran et al., 2015; İlgar et al., 2021; Onoda et al., 2023), and CYP90B50 is considered the first committed enzyme in the pathway from cholesterol to diosgenin (Christ et al., 2019; Zhou et al., 2022). In addition, CAS catalyzes the first committed step from 2,3-oxidosqualene to cholesterol in plants (Bach, 2017). Previous studies have shown that the treatments with MeJA or ethylene elevate *CAS* expression, which correlates with increased diosgenin biosynthesis (Diarra et al., 2013; Javan et al., 2024). Thus, these findings suggest that *TfWRKY40* may regulate diosgenin biosynthesis primarily by transcriptionally modulating expression of *HMGR1*, *CAS1* or *CYP90B50*.

## 4 Discussion

MeJA is a well-established signaling molecule commonly used to stimulate the production of plant secondary metabolites. Previous studies have demonstrated that MeJA treatment enhances diosgenin biosynthesis in *T. foenum-graecum* (Debjani and Bratati, 2011; Chaudhary et al., 2015; Ciura et al., 2017a, 2017b; Irankhah et al., 2020; Gonda et al., 2023). In our study, application of 0.015% (v/v) MeJA to *T. foenum-graecum* seedlings significantly increased diosgenin accumulation, in agreement with these prior findings (Zhou et al., 2019). During the early phase of induction (6–12 h), diosgenin levels rose markedly (**Figure 1A**), indicating rapid transcriptional activation of biosynthetic genes and efficient channeling of metabolic flux, hallmarks of a typical saponin-type defense response in plants. However, at 24–48 h post-treatment, no significant difference was observed between the MeJA-treated and control groups (**Figure 1A**), possibly reflecting a temporary metabolic adaptation or the resolution of the acute MeJA-induced response. Interestingly, by 72 h, diosgenin levels in treated seedlings again showed a slight yet consistent upward trend compared to controls, although the magnitude of this increase was notably lower than during the initial induction phase (**Figure 1A**). These temporal fluctuations are consistent with the findings previously described by Zhou et al. (2019), who also reported non-linear patterns of MeJA-induced diosgenin accumulation in *T. foenum-graecum*. Although the timing and extent of these fluctuations observed in this study differed from those reported by Zhou et al. (2019), likely due to variation in cultivars and growth conditions, the overall trend of transient enhancement followed by stabilization appears to be a common regulatory feature. This dynamic response may reflect a broader metabolic reprogramming during the treatment: early resource allocation favors rapid diosgenin production as a frontline stress response, while a longer-term adaptation to MeJA stress could involve a shift toward alternative defense mechanisms, leading to a reduced investment in diosgenin biosynthesis. Thus, although the MeJA-treated seedlings maintained higher diosgenin levels than the controls at 72 h, the increment in diosgenin content was attenuated compared to the increase observed at 6–12 h, suggesting a shift in physiological priorities as the induced defense response progressed.

In *T. foenum-graecum*, diosgenin primarily exists as a glycosylated form (e.g., dioscin), a steroidal saponin that, like most triterpenoid glycosides, accumulates predominantly in the leaves, with significantly lower concentrations detected in stems and roots (**Figure 1C**). This tissue-specific distribution pattern closely mirrors the organ-specific expression profile of *TfWRKY40*, suggesting a potential transcriptional regulatory relationship between *TfWRKY40* and diosgenin biosynthesis (Brenac and Sauvaire, 1996; Ortuño et al., 1998; Oncina et al., 2000). In contrast, the expression patterns of two other candidate genes, DN12554 and DN20355, showed poor correlation with diosgenin accumulation (**Figure 1D**), further supporting *TfWRKY40* (DN18092) as the most likely transcriptional regulator involved in the regulation of diosgenin biosynthesis in *T. foenum-graecum*. Nevertheless, it remains unclear whether diosgenin is synthesized exclusively in the leaves or also in other tissues and subsequently transported. If inter-tissue transport does occur, *DN12554* and *DN20355* may also play roles in regulating diosgenin biosynthesis elsewhere and thus warrant further investigation. Future studies involving isotope labeling or tissue-specific metabolic flux analysis will be essential to determine the spatial origin of diosgenin biosynthesis.

*In planta* experiments conducted in *T. foenum-graecum* hairy roots demonstrated that overexpression of *TfWRKY40* significantly increased diosgenin accumulation, whereas its silencing via RNAi led to a marked reduction in diosgenin levels (**Figure 4**). This positive correlation between *TfWRKY40* expression and diosgenin content strongly supports its role as a transcriptional activator in diosgenin biosynthesis. Transcriptomic analysis further revealed that overexpression and RNAi-mediated silencing of *TfWRKY40* resulted in significant upregulation and downregulation of key enzymatic genes involved in diosgenin biosynthesis, notably HMGR (a rate-limiting enzyme in the MVA pathway), CAS (a critical enzyme in cholesterol biosynthesis) and CYP90B50 (a key rate-limiting enzyme in the biosynthetic pathway from cholesterol to diosgenin) (**Figure 5**), indicating that *TfWRKY40* regulates diosgenin accumulation by transcriptionally activating cholesterol biosynthesis and conversion of cholesterol to diosgenin. This discovery suggests that *TfWRKY40* modulates diosgenin biosynthesis by exerting transcriptional control through the pathways both upstream and downstream of cholesterol, likely by regulating cholesterol precursor supply and possibly by also influencing the downstream conversion of cholesterol to diosgenin. Given that intracellular cholesterol availability directly determines the metabolic flux toward diosgenin (Joly et al., 1969a; 1969b; Stohs et al., 1969; Christ et al., 2019; Zhou et al., 2022), *TfWRKY40* appears to play a central role in coordinating this pathway, linking the upstream metabolic regulation with the downstream secondary metabolite production.

Previous studies have demonstrated that MeJA-induced accumulation of diosgenin in *T. foenum-graecum* is associated with the upregulation of HMGR and sterol-3-glucosyltransferase (STRL) (Chaudhary et al., 2015). More recently, transcriptomic analyses have shown that MeJA treatment also enhances the expression of other key enzymes in the diosgenin biosynthetic pathway, including SQS, SQE, and CAS (Javan et al., 2024). The central role of *CAS* in this pathway has been further corroborated by Ciura et al. (2017a); Ciura et al. (2018), who identified it as a crucial regulatory node. Supporting this, Diarra et al. (2013) reported that ethylene treatment specifically elevated *CAS* expression without affecting the expression of *SQS* or *FPPS*, which was accompanied by an increase of diosgenin, highlighting the unique and pivotal function of *CAS* in directing metabolic flux toward diosgenin biosynthesis.

The coordinated upregulation of *TfWRKY40* expression during MeJA-induced diosgenin accumulation in *T. foenum-graecum*, together with the marked downregulation or upregulation of *CAS1*, *HMGR1* or *CYP90B50* in *TfWRKY40*-RNAi or *TfWRKY40*-OE transgenic hairy roots, strongly suggests that *TfWRKY40* regulates diosgenin biosynthesis likely through direct transcriptional control of *CAS1, HMGR1* and/or *CYP90B50*. Notably, only one of the three *HMGR* transcripts, *HMGR1*, showed a significant correlation with *TfWRKY40* expression, whereas *HMGR2* and *HMGR3* did not display similar expression patterns. A comparable expression pattern was observed for *SMO4*, where only *SMO4-3* exhibited consistent expression changes in response to *TfWRKY40* manipulation, indicating potential transcript-level functional divergence among gene family members. Therefore, the observed correlations between *TfWRKY40* and *HMGR1*/*SMO4-3* warrant further isoform-specific functional validation to confirm these regulatory relationships. We hypothesize that *TfWRKY40* binds to the promoter region of the *CAS1*, *HMGR1* or *CYP90B50* gene, thereby modulating its transcription and enhancing metabolic flux through the post-2,3-oxidosqualene steps of diosgenin biosynthetic pathway. To substantiate this proposed regulatory mechanism, it would be of particular interest to confirm these interactions experimentally using chromatin immunoprecipitation (ChIP) and electrophoretic mobility shift assay (EMSA) when the nuclear genome sequence of *T. foenum-graecum* becomes available.

## 5 Conclusion

In this study, we demonstrated that MeJA treatment significantly enhanced diosgenin production in *Trigonella foenum-graecum* seedlings, with a pronounced increase observed at 12 hours post-induction, thereby confirming the positive regulatory role of MeJA in diosgenin biosynthesis. Through a comparative transcriptome analysis, we identified *Tf*WRKY40, a WRKY family transcription factor that showed expression pattern correlated to diosgenin accumulation. Utilizing the *T. foenum-graecum* hairy root transformation, we functionally validated *Tf*WRKY40 as a key positive regulator of diosgenin biosynthesis *in vivo*. Transcriptomic analysis of the *TfWRKY40*-RNAi and *TfWRKY40*-OE transgenic hairy roots, along with their respective empty vector-transformed controls, revealed that *TfWRKY40* likely promotes diosgenin biosynthesis by transcriptionally regulating expression of the key biosynthetic genes or transcript variants, particularly *CAS1*, *HMGR1*, and *CYP90B50*. These genes or transcript variants are essential for diosgenin biosynthesis from the upstream to downstream steps of cholesterol metabolism, with cholesterol serving as a critical precursor in the pathway.

## Data Availability

The transcriptome data have been deposited in the NCBI Sequence Read Archive (SRA) under BioProject accession number PRJNA1321096. The nucleotide sequence of *TfWRKY40* has been submitted to GenBank with accession number PX310700.
