# Supplementary material for: TfWRKY40 positively regulates diosgenin biosynthesis in Trigonella foenum-graecum L
Source: Front Plant Sci. 2025 Sep 24;16:1666107. doi: 10.3389/fpls.2025.1666107 (PMC12504280; doi:10.3389/fpls.2025.1666107)
Supplement: Supplementary file 1 [file DataSheet1.docx]

Supplementary Figures

***TfWRKY40* positively regulates diosgenin biosynthesis in *Trigonella foenum-graecum* L.**

**Chuanjia Xu^1,2#^, Nan Tang^2^, Yehan Xu^2#^, Changfu Li^2^, Yansheng Zhang^1,2*^**

^1^School of Environmental and Chemical Engineering, Shanghai University, Shanghai 200444, China

^2^ Shanghai Key Laboratory of Bio-Energy Crops, Synthetic Biology Research Center, School of Life Sciences, Shanghai University, Shanghai, 200444, China

# These authors contributed equally to this study

*** Correspondence:**Yansheng Zhang

Email: zhangys1@shu.edu.cn


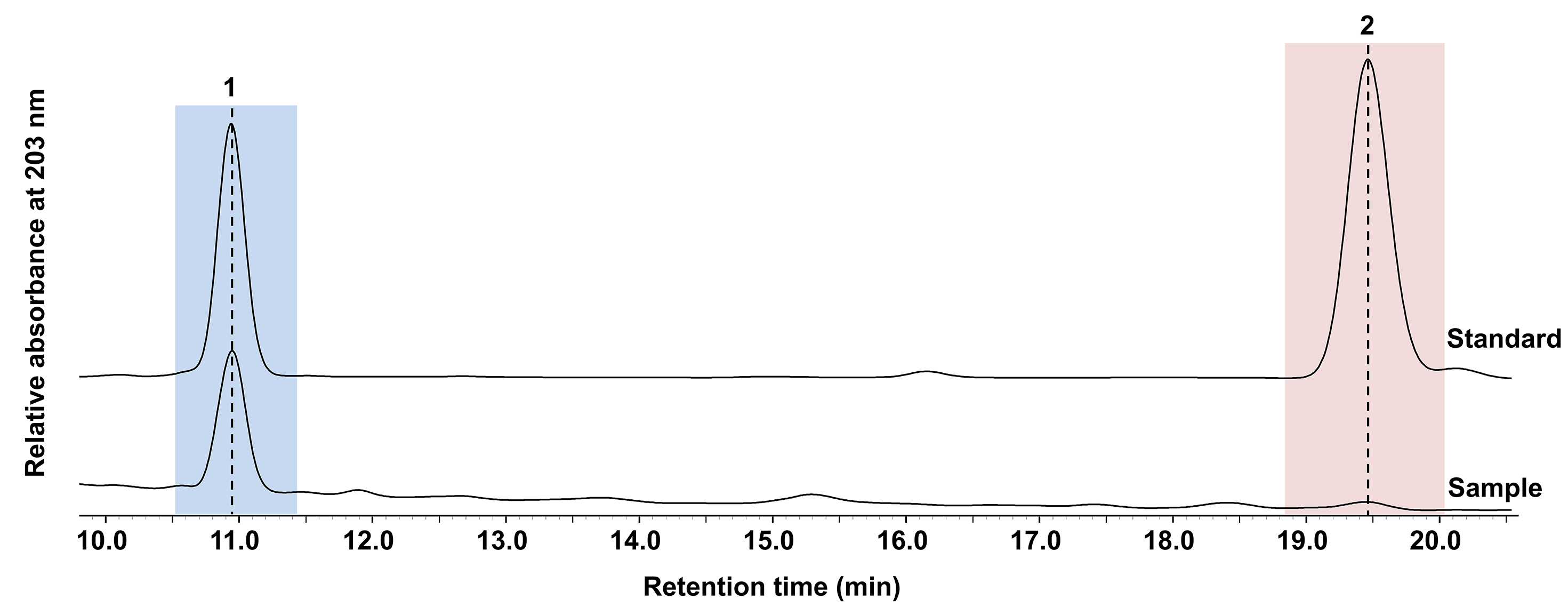


**Supplementary Figure S1** Representative HPLC chromatograms of ursolic acid and diosgenin in chemical standards and sample extracts. The retention times of ursolic acid (peak 1, 10.93 min) and diosgenin (peak2, 19.47 min) in the sample matched those of the corresponding standards, confirming their presence in the extract.


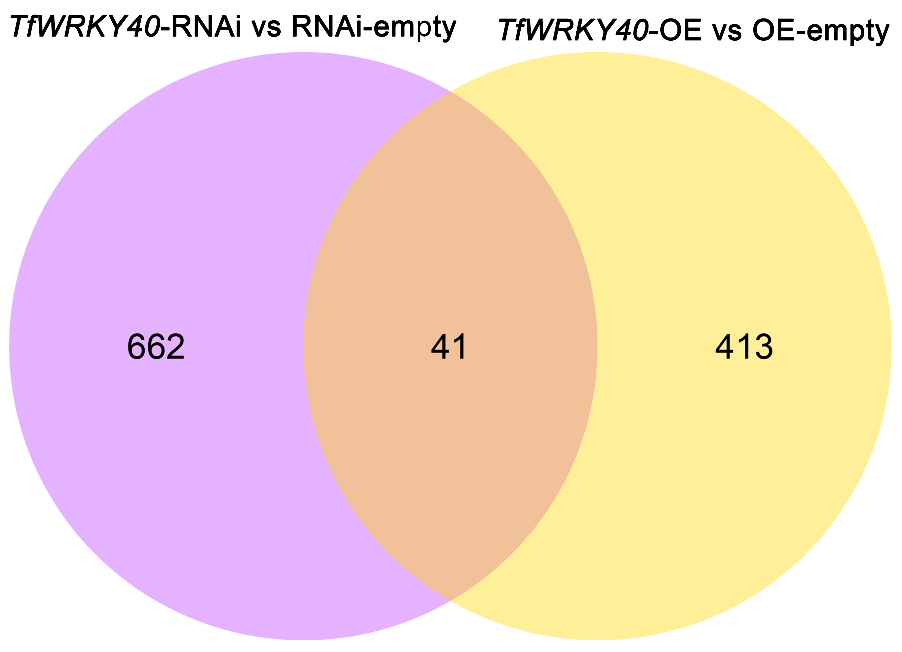


**Supplementary Figure S2.** Venn diagram of differentially expressed genes (DEGs) identified from comparisons between *TfWRKY40*-RNAi and RNAi-empty hairy roots, as well as between *TfWRKY40*-OE and OE-empty hairy roots.


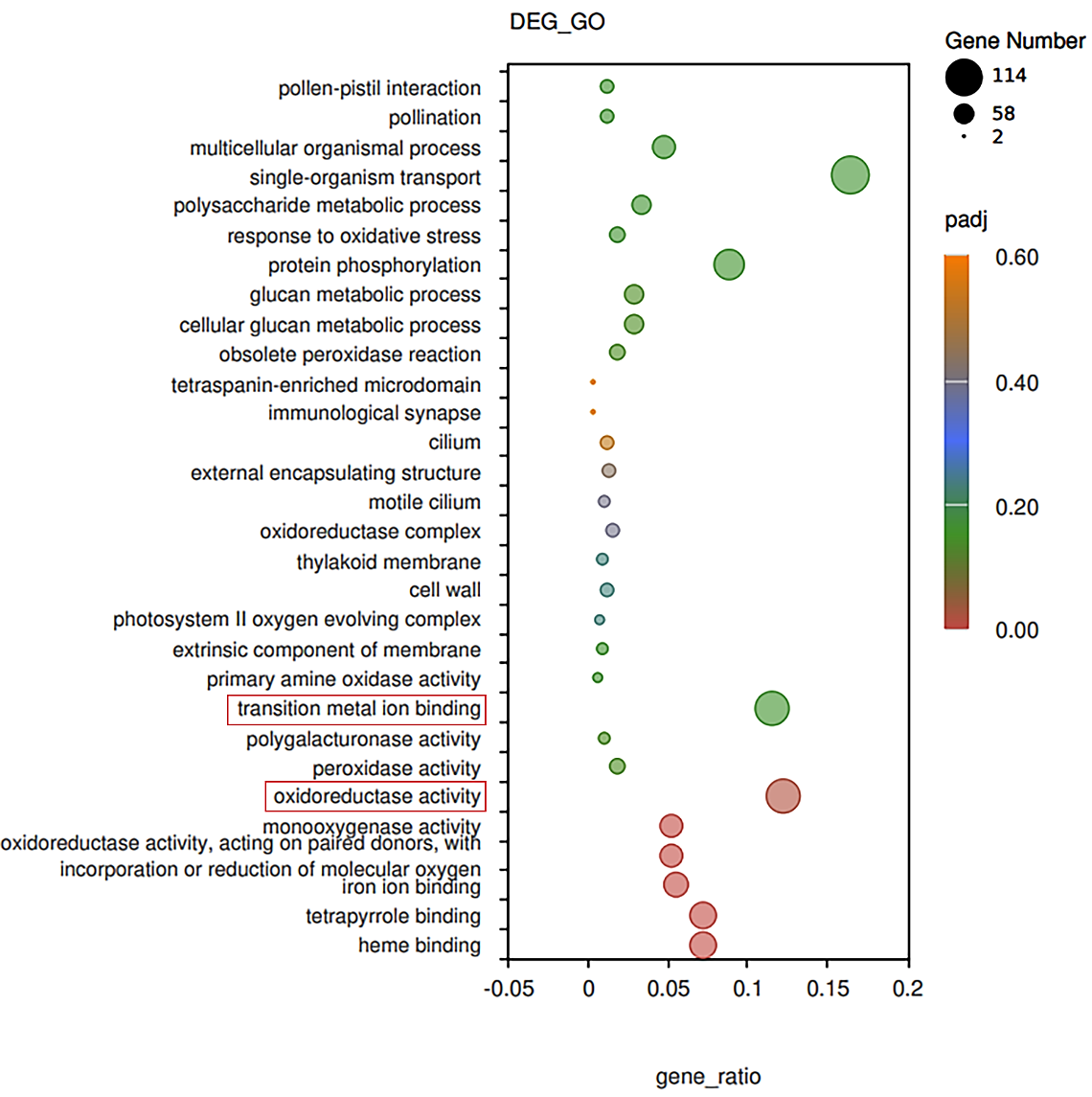


**Supplementary Figure S3.** Gene Ontology (GO) enrichment analysis of the DEGs from *TfWRKY40*-overexpressing or -silenced *Trigonella foenum-graecum* hairy roots in comparison with their empty-vector transformed controls.


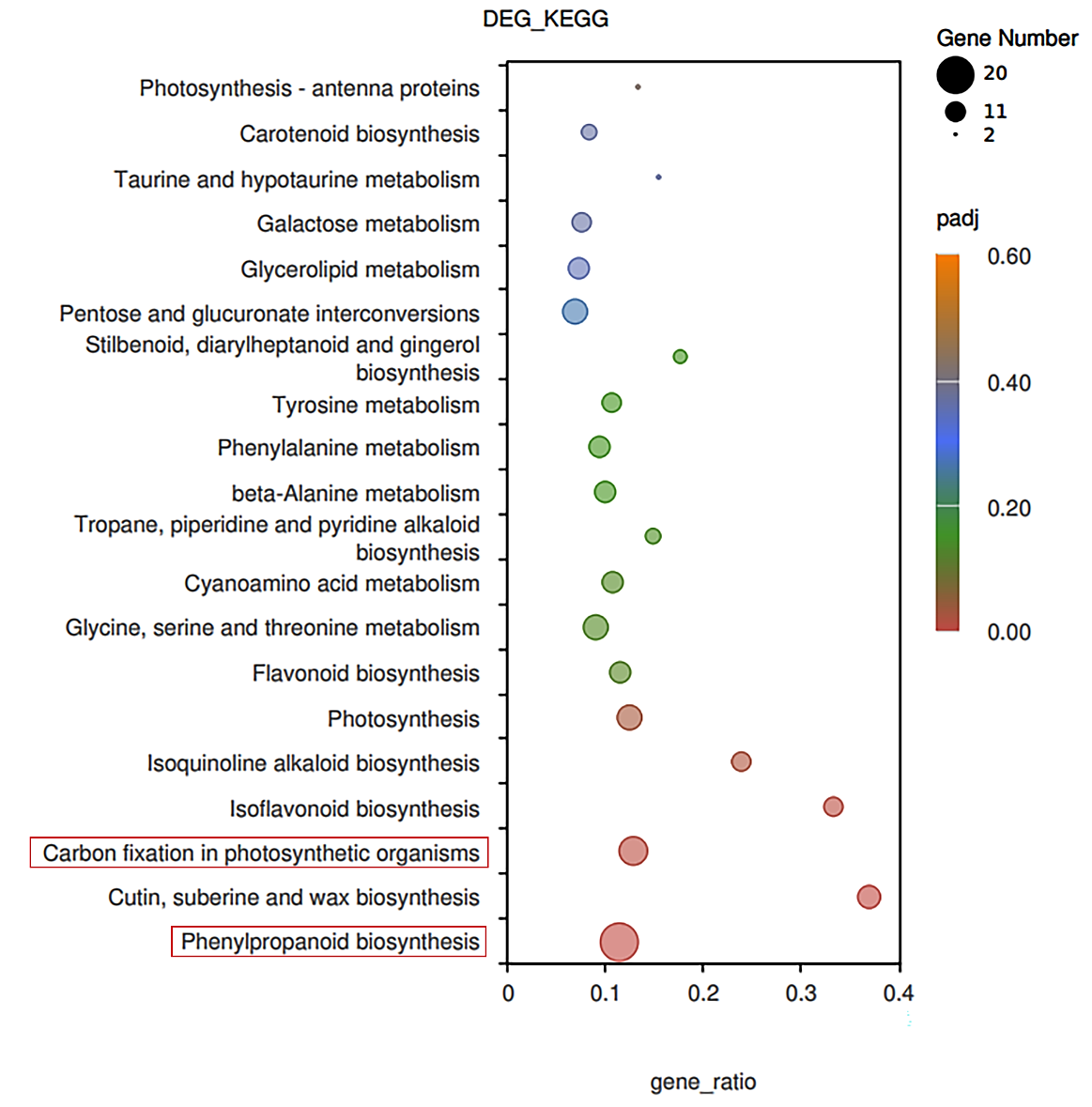


**Supplementary Figure S4** Kyoto Encyclopedia of Genes and Genomes (KEGG) pathway enrichment analysis of the DEGs identified from transcriptomic comparisons of the *Trigonella foenum-graecum* hairy roots overexpressing or silencing *TfWRKY40*, relative to their respective controls.
